# Structural and Serological Studies of the O6-Related Antigen of *Aeromonas veronii* bv. *sobria* Strain K557 Isolated from *Cyprinus carpio* on a Polish Fish Farm, which Contains l-perosamine (4-amino-4,6-dideoxy-l-mannose), a Unique Sugar Characteristic for *Aeromonas* Serogroup O6

**DOI:** 10.3390/md17070399

**Published:** 2019-07-05

**Authors:** Katarzyna Dworaczek, Dominika Drzewiecka, Agnieszka Pękala-Safińska, Anna Turska-Szewczuk

**Affiliations:** 1 Department of Genetics and Microbiology, Maria Curie-Skłodowska University in Lublin, Akademicka 19, 20-033 Lublin, Poland; 2 Laboratory of General Microbiology, Department of Biology of Bacteria, Faculty of Biology and Environmental Protection, University of Łódź, Banacha 12/16, 90-237 Łódź, Poland; 3 Department of Fish Diseases, National Veterinary Research Institute, Partyzantów 57, 24-100 Puławy, Poland

**Keywords:** *Aeromonas*, fish pathogen, lipopolysaccharide (LPS), structure, O-antigen, O-polysaccharide, l-perosamine, immunospecificity, NMR spectroscopy, mass spectrometry

## Abstract

Amongst *Aeromonas* spp. strains that are pathogenic to fish in Polish aquacultures, serogroup O6 was one of the five most commonly identified immunotypes especially among carp isolates. Here, we report immunochemical studies of the lipopolysaccharide (LPS) including the O-specific polysaccharide (O-antigen) of *A. veronii* bv. *sobria* strain K557, serogroup O6, isolated from a common carp during an outbreak of motile aeromonad septicemia (MAS) on a Polish fish farm. The O-polysaccharide was obtained by mild acid degradation of the LPS and studied by chemical analyses, mass spectrometry, and ^1^H and ^13^C NMR spectroscopy. It was revealed that the O-antigen was composed of two O-polysaccharides, both containing a unique sugar 4-amino-4,6-dideoxy-l-mannose (*N*-acetyl-l-perosamine, l-Rha*p*4NAc). The following structures of the O-polysaccharides (O-PS 1 and O-PS 2) were established: O-PS 1: →2)-α-l-Rha*p*4NAc-(1→; O-PS 2: →2)-α-l-Rha*p*4NAc-(1→3)-α-l-Rha*p*4NAc-(1→3)-α-l-Rha*p*4NAc-(1→. Western blotting and an enzyme-linked immunosorbent assay (ELISA) showed that the cross-reactivity between the LPS of *A. veronii* bv. *sobria* K557 and the *A. hydrophila* JCM 3968 O6 antiserum, and *vice versa*, is caused by the occurrence of common α-l-Rha*p*4NAc-(1→2)-α-l-Rha*p*4NAc and α-l-Rha*p*4NAc-(1→3)-α-l-Rha*p*4NAc disaccharides, whereas an additional →4)-α-d-Gal*p*NAc-associated epitope defines the specificity of the O6 reference antiserum. Investigations of the serological and structural similarities and differences in the O-antigens provide knowledge of the immunospecificity of *Aeromonas* bacteria and are relevant in epidemiological studies and for the elucidation of the routes of transmission and relationships with pathogenicity.

## 1. Introduction

The genus *Aeromonas*, which belongs to the family *Aeromonadaceae* along with four other genera *Telumonas*, *Oceanimonas*, *Oceanisphaera* and *Zobellella*, is composed of a large number of species classified within 17 DNA-hybridization groups (HG) or genomospecies, and 14 phenospecies [1,2,3,4,5]. These Gram-negative rods are typically found in aquatic environments, e.g., freshwater, estuarine and coastal water, seawater, drinking water supplies, polluted waters, marine, and freshwater sediment and sand. Aeromonads have also been isolated from animals, food, and various clinical samples. Most frequently, *Aeromonas* spp. strains are pathogenic to poikilothermic animals including amphibians, fish, and reptiles. They also can be associated with infections of birds and mammals [4,5,6]. In fish, non-motile psychrophilic species of *Aeromonas salmonicida* subsp. salmonicida are pathogenic to Salmonidae*,* provoking systemic furunculosis [7,8]. In turn, the representatives of *Aeromonas hydrophila*, *Aeromonas bestiarum*, *Aeromonas salmonicida*, *Aeromonas jandaei*, and *Aeromonas veronii* bv. *sobria* have been described as causative agents of diseases in a variety of fish species. These include motile Aeromonas septicemia (MAS), clinical conditions associated with systemic infection resulting in high mortality rates and severe economic losses in aquacultures, and a chronic type of disease called motile Aeromonas infection (MAI) causing erosion of fins, skin lesions, and ulcerations [9,10,11,12]. The mesophilic and motile *A. veronii*, *A. hydrophila*, *A. caviae* and *A. schubertii* species are potentially pathogenic to humans. Immunocompromised patients and children are especially vulnerable. Clinical presentations include gastroenteritis, wound infections, biliary tract infections, pneumonia, meningitis, septic arthritis, or septicemia without an obvious focus of infection [4,13,14,15,16]. 

The pathogenicity of *Aeromonas* is determined by their ability to produce extracellularly secreted enzymes and toxins i.e., hemolysins, cytotonic and cytotoxic enterotoxins, proteases, lipases, gelatinases, and leucocidins, which play an important role in both the initial steps and spread of the infection process. Moreover, cell-surface constituents, including outer membrane proteins, S-layers, surface polysaccharides (capsule, lipopolysaccharide, and glucan), flagella, and pili, have been identified as important compounds associated with *Aeromonas* virulence [4,10,17,18,19]. 

Lipopolysaccharide (LPS) is the dominant glycolipid in the outer leaflet of the outer membrane of Gram-negative bacteria, which mediates their virulence. The high-molecular-weight S-LPS glycoforms have a tripartite structure comprising lipid A, core oligosaccharide, which together with lipid A contributes to maintenance of the integrity of the outer membrane, and the O-specific polysaccharide (O-PS), which is connected to the core and most frequently consists of a heteropolymer composed of repeating oligosaccharide units [20,21,22,23]. The O-specific polysaccharide is a surface antigen called the somatic O-antigen, whose high degree of diversity determines the specificity of each bacterium and gives the basis for their serological classification. Serotyping to identify bacterial strains is invaluable for epidemiological investigations since many O-serotypes are associated with specific disease syndromes [21,24]. 

*Aeromonas* strains are serologically heterogeneous and they have been characterized and classified based on O-antigens into 44 serogroups using the NIH scheme (National Institute of Health, Japan) proposed by Sakazaki and Shimada [25], which can be further extended to 97 O serogroups after inclusion of provisional new serogroups [26]. The variants of the O-specific polysaccharide, which represents a specific antigenic fingerprint for bacteria, might be very useful, e.g., for identification of *Aeromonas* strains eliciting infections in farmed fish and for diagnosis of etiological agents of gastrointestinal infections in humans. 

Despite the large antigenic diversity of *Aeromonas*, only several serogroups i.e., O11, O16, O18, and O34 (NIH scheme) have been reported, most frequently, as predominating especially in clinical specimens. These O-serotypes of mesophilic *Aeromonas* were associated with most cases of bacteremia, suggesting their role in the pathogenesis of some systemic diseases [4]. 

As shown in previous reports, the distribution of the serogroups of *Aeromonas* strains may be related to geographic location [27,28]. Such differences in the occurrence of *Aeromonas* species serogroups were associated with outbreaks of septicemia in fish. Strains belonging to serogroup O14 have been identified as pathogens of European eels [29]. As revealed in recently published studies by Kozińska and Pękala [28], the majority of isolates that are pathogenic to carp in Polish aquacultures represented serogroups O3, O6, O41, PGO4, and PGO6, whereas serogroups O11, O16, O18, O33, PGO1, and PGO2 dominated among both carp and trout isolates. In turn, motile aeromonas septicemia (MAS) incidences in rainbow trout have been related to the strains of serogroups O11, O16, O34, and O14. Moreover, pathogenic isolates of two species: *Aeromonas veronii* bv. *sobria* and *Aeromonas sobria* were mainly classified within the O6 serogroup. 

The *A. veronii* species, originally described by Hickman-Brenner et al. [30] as a novel member of the genus, is commonly associated with diarrhea. Nevertheless, this species, which consists of two biovars, *A. veronii* bv. *sobria*, which is negative for aesculin hydrolysis and ornithine decarboxylase, and *A. veronii* bv. *veronii*, which is positive for these reactions [15,31], is commonly known as a fish pathogen associated mainly with ulcerative syndrome [32,33]. It is worth emphasizing that *A. veroni* bv. *sobria* was one of the dominant species among carp isolates collected during 5 years in Polish culture facilities, and 76% of these isolates were classified as virulent [28]. In the light of the increased *Aeromonas* infection incidence rate and the economic importance of these diseases in cultured fish, it is essential to characterize virulence factors associated with the pathogenesis of this species, particularly including LPS, which is the major surface glycoconjugate of Gram-negative bacteria. Recently, two new structures of O-antigens were established for the species *A. veronii* bv. *sobria* and *A. sobria* [34,35]. 

Here we report immunochemical investigation of LPS, especially the O-specific polysaccharide of *A. veronii* bv. *sobria* strain K557, which was isolated from the common carp (*Cyprinus carpio* L.) during an outbreak of motile aeromonad infection/motile aeromonad septicemia (MAI/MAS) on a Polish fish farm [28,36]. The structural characterization revealed that the O-antigen was composed of two O-polysaccharides, both containing a unique sugar 4-amino-4,6-dideoxy-l-mannose (*N*-acetyl-l-perosamine, l-Rha*p*4NAc). Serological studies using Western blotting and an enzyme-linked immunosorbent assay (ELISA) with intact and adsorbed O-antisera showed that the O-antigen of *A. veronii* bv. *sobria* strain K557 is related but not identical to that of *A. hydrophila* JCM 3968 O6, which is a reference strain for *Aeromonas* serogroup O6 [37]. 

## 2. Results

### 2.1. Bacterial Cultivation, Isolation of LPS, and SDS-PAGE Study

**Figure 1:**
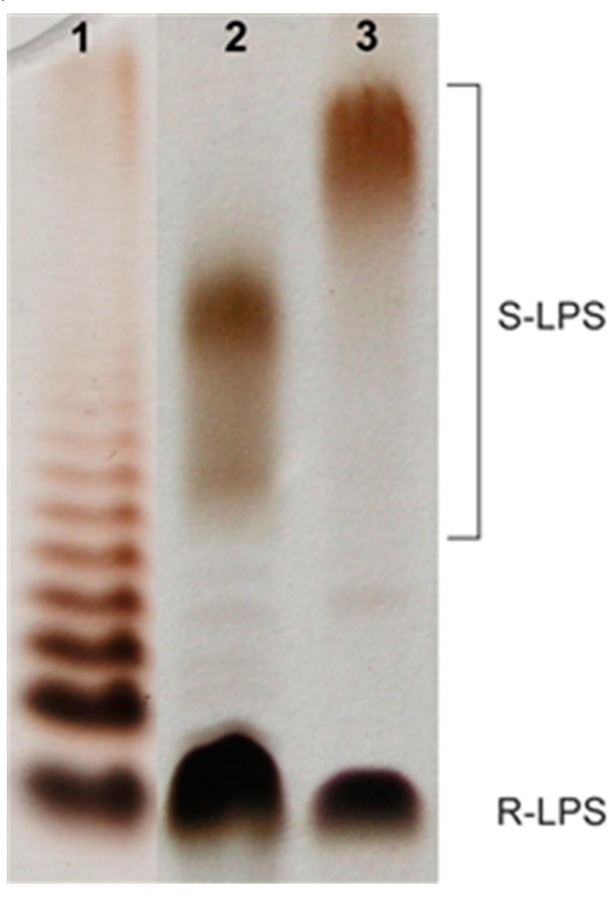
Silver-stained SDS-PAGE of the LPS of *A. veronii* bv. *sobria* strain K557 (lane 2, 2 μg), *A. hydrophila* JCM 3968, O6 (lane 3, 2 μg), and *Salmonella enterica* sv. Typhimurium (Sigma-Aldrich, St. Louis, MO, USA) as a reference (lane 1, 2 μg). R-LPS and S-LPS species are indicated.

Cells of *Aeromonas veronii* bv. *sobria* strain K557 were extracted with hot aqueous 45% phenol [38], and LPS species were harvested from the phenol phase in a yield of 3.9% of the bacterial cell mass. SDS-PAGE analysis of the LPS followed by silver staining showed a pattern typical for LPS isolated from smooth bacterial cells (Figure 1) with the content of both slow-migrating high-molecular-weight (HMW) S-LPS species and fast-migrating low-molecular-weight R-LPS glycoforms. Moreover, the electrophoregram indicated that the studied S-LPS contained molecules where the core oligosaccharides were substituted with shorter O-chains in comparison to the LPS species of *A. hydrophila* JCM 3968, O6. 

### 2.2. Serological Studies of the A. veronii bv. sobria K557 O-antigen

*A. veronii* bv*. sobria* strain K557 was serologically typed by agglutination tests using heat-inactivated bacteria and antisera for 44 defined *Aeromonas* O-serogroups (NIH system) and 20 provisional serogroups (PGO1–PGO20) for selected Polish isolates and classified as a representative of the *Aeromonas* serogroup O6 [28,36]. 

The LPS preparations from *A. veronii* bv*. sobria* strain K557 and *A. hydrophila* JCM 3968, which is the reference strain to serogroup O6, were studied by Western blotting and ELISA with intact and adsorbed polyclonal rabbit O-antisera. 

**Figure 2:**
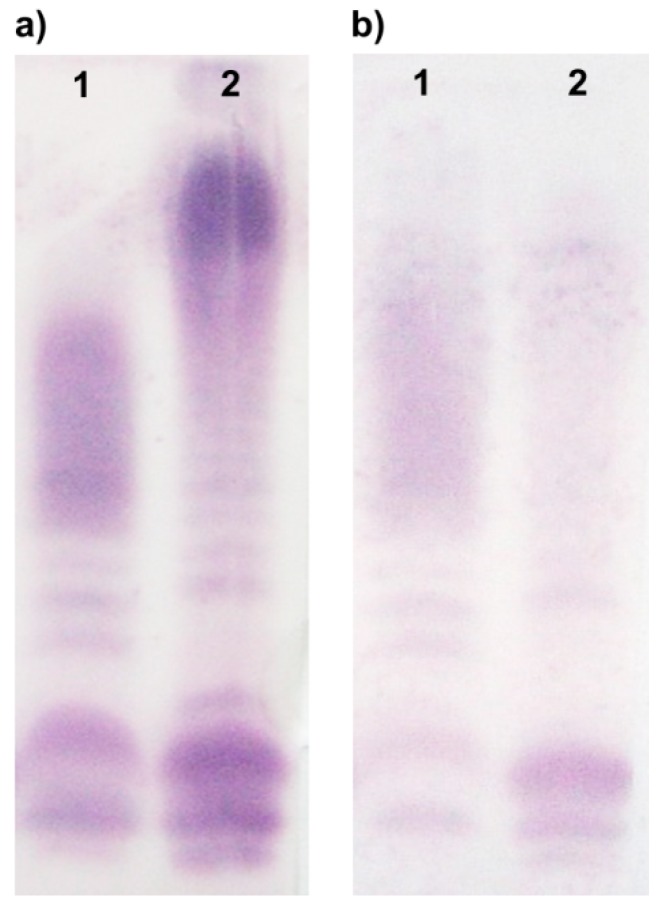
Western blots of lipopolysaccharides after SDS-PAGE with the intact reference O6 (**a**) and anti-K557 O-antisera (**b**). Lanes: 1, LPS of *A. veronii* bv*. sobria* strain K557; 2, LPS of *A. hydrophila* strain JCM 3968.

In Western blot, the reference antiserum O6 recognized electrophoretically separated LPS molecules of both strains (Figure 2a). Strong reaction to slow-migrating bands corresponding to the O-PS containing LPS species was observed and suggested that the O-antigens shared common epitopes. Additionally, the stained bands of fast-migrating R-LPS molecules may indicate similarities in the core region of the studied strains. In turn, the other Western blot (Figure 2b) with *A. veronii* bv. *sobria* K557 O-antiserum revealed the recognition of antigenic determinants within LPS molecules of both *A. veronii* bv*. sobria* K557 and *A. hydrophila* JCM 3968 strains; however, the cross-reaction with the heterologous LPS was weaker than in the homologous system. 

Accordingly, in ELISA (Table 1), the rabbit polyclonal O antiserum specific for *A. hydrophila* JCM 3968 O6 reacted strongly with the homologous LPS, and cross-reaction was observed with the LPS of *A. veronii* bv*. sobria* strain K557 to the lower titer of 1:64,000. In turn, the polyclonal O-antiserum against *A. veronii* bv*. sobria* strain K557 revealed reactions at the same level as both the homologous and heterologous LPS samples. The O6 reference antiserum revealed stronger reactivity than the K557-specific one, as demonstrated by the Western blotting results. 

Adsorption of the antiserum specific for the *A. veronii* bv*. sobria* K557 O-antigen with *A. hydrophila* strain JCM 3968 O6 cells totally abolished the reactivity of this antiserum with both LPS preparations. The opposite reaction, i.e., adsorption of the reference O6 antiserum with the *A. veronii* bv*. sobria* K557 cells, only decreased its reactivity in the homologous system, whereas there was no reaction of the adsorbed O6 antiserum with *A. veronii* bv*. sobria* K557 LPS. The latter findings indicated that the adsorption process was complete and resulted in the removal of anti-K557 antibodies from the reference O6 antiserum. The remaining antibodies, strongly reacting with the homologous O6 LPS, were most probably specific to an additional epitope characteristic for the *A. hydrophila* JCM 3968 O6 antigen. 

These data indicated that the reference O6 antiserum and the *A. veronii* bv*. sobria* K557 O-antiserum shared common antibodies but the reference one contained additional immunoglobulins recognizing structural determinants that were not present in the *A. veronii* bv*. sobria* K557 O-antigen. 

Therefore, detailed chemical analyses were performed to establish the structure of the *A. veronii* bv*. sobria* strain K557 O-PS. 

### 2.3. Chemical and Mass Spectrometry Analyses of LPS

Compositional analysis of the degraded polysaccharide (dgPS) liberated from the phenol-soluble LPS was performed using GLC-MS of alditol acetates. It showed the presence of d-glucose (d-Glc), d-galactose (d-Gal), 2-amino-2-deoxy-d-glucose (d-GlcN), d-*glycero*-d-*manno*-heptose (d,d-Hep), and l-*glycero*-d-*manno*-heptose (l,d-Hep) in a ratio of 1.0 : 1.2 : 1.3 : 1.5 : 3.8 as the core oligosaccharide components. The chemical analysis also revealed 6-deoxymannose (Rha), 2-amino-2,6-dideoxyglucose (QuiN), and 4-amino-4,6-dideoxyhexose (identified as Rha4N, see Section 2.4.). Rha4N was found as the main component of the O-polysaccharide part (see Section 2.4.). Kdo (3-deoxy-d-*manno*-2-octulosonic acid)—the only acidic sugar—was found in the LPS after treatment of the LPS with 48% aqueous HF (hydrofluoric acid), which suggested its phosphorylation [37,39,40]. The GLC-MS analysis of fatty acids as methyl esters and *O*-TMS derivatives revealed that 3-hydroxytetradecanoic [14:0(3-OH)], 3-hydroxy*iso*pentadecanoic [*i*15:0(3-OH)], as well as dodecanoic (12:0) and tetradecanoic (14:0) acids, were the most abundant species in a ratio of 6.3 : 2.4 : 1.5 : 1.0. GlcN was identified as the sugar component of lipid A. 

**Figure 3:**
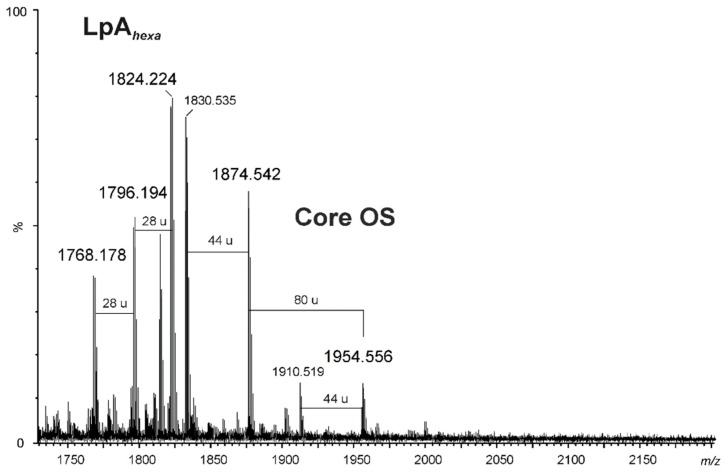
MALDI-TOF mass spectrum (negative ion mode) of the LPS of *A. veronii* bv. *sobria* strain K557. The notations indicate: 28 u—differences in (CH_2_)_2_ in the fatty acid chain length; 44 u—loss of CO_2_; 80 u—loss of phosphate; LpA*_hexa_* - hexaacylated lipid A; Core OS—core oligosaccharide.

The negative ion matrix-assisted laser desorption/ionization time-of-flight (MALDI-TOF) mass spectrum of the *A. veronii* bv*. sobria* K557 lipopolysaccharide (Figure 3) showed the most intensive signals in the *m*/*z* range 1600–2000, which were attributable to a lipid A and a core oligosaccharide species (Y- and C-type fragment ions) arising from an in-source fragmentation at the glycosidic bond between the Kdo and the lipid A [41]. The ions at *m*/*z* 1768.17, 1796.19, and 1824.22 originated from hexaacylated lipid A species (Y-fragment ions) [37]. The ion at *m*/*z* 1824.22 represented a variant of lipid A, where the diglucosaminyl backbone bisphosphorylated at O-1 and O-4’ was substituted by four 3-hydroxytetradecanoic acids 14:0(3-OH) and two tetradecanoic acids 14:0, instead of two dodecanoic acids 12:0, compared with the ion at *m*/*z* 1768.17. In turn, the ion at *m*/*z* 1796.19 contained a sugar backbone acylated by three, instead of four, 3-hydroxytetradecanoic acids, one 3-hydroxy*iso*pentadecanoic acid, and two dodecanoic acids. The composition of the ions is shown in Table 2. 

The ions at *m*/*z* 1954.5 and 1874.5 (C-fragment ions) were assigned to the core oligosaccharide with the following composition: Hep_6_Hex_2_HexN_1_Kdo. The mass difference between the ions of 80 amu corresponded to species containing phosphorylated and dephosphorylated variants of the core oligosaccharide, respectively. 

### 2.4. Structural Studies of O-polysaccharide (O-PS)

The O-PS was released from phenol-soluble LPS by mild-acid degradation followed by gel-permeation-chromatography (GPC) on Sephadex G-50 fine to give a high-molecular-weight O-polysaccharide with the yield of 33% of the LPS mass. GLC-MS sugar analysis of alditol acetates obtained after full acid hydrolysis of the O-PS showed the presence of rhamnose and 4-amino-4,6-dideoxymannose (Rha4N) in a peak area ratio ~1: 28.4. Other compounds detected in a small amount (below 10 %) in the GLC chromatogram of the *A. veronii* bv. *sobria* K557 O-PS, i.e., Glc, Gal and two heptose isomers (d,d-Hep and l,d-Hep), represented the core oligosaccharide sugars 

Determination of the absolute configurations of the monosaccharides by GLC of the acetylated (*S*)- and (*SR*)-2-octyl glycosides [42] showed that Rha4N had the l configuration. Samples from the O-polysaccharide of *Citrobacter gillenii* O9a,9b [43] and the O-PS of *A. hydrophila* JCM 3968 [37] were used as a reference standard of d-Rha4N and l-Rha4N, respectively. 

**Figure 4:**
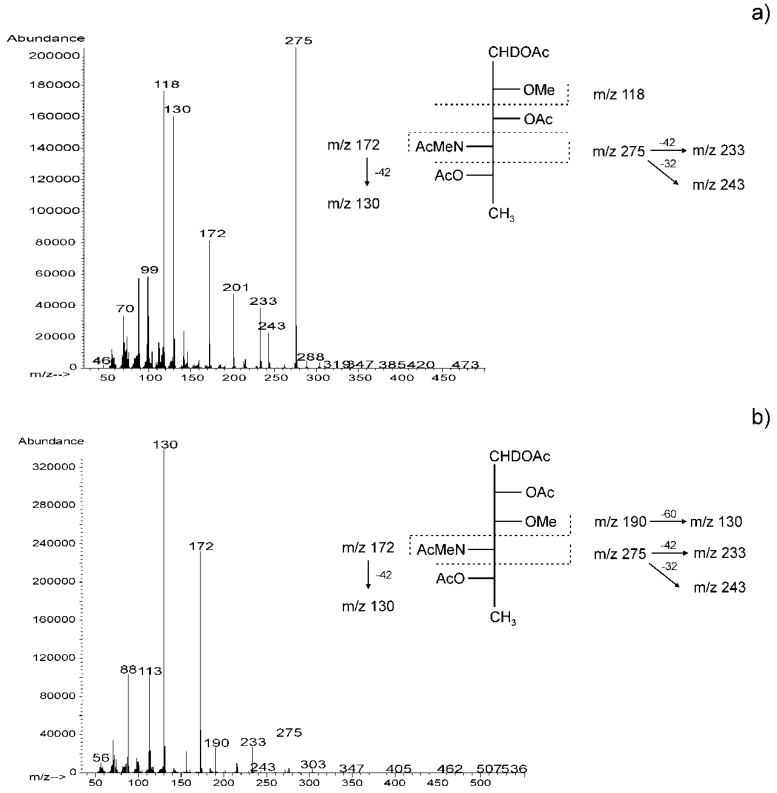
*EI* (electron impact) mass spectra and fragmentation pathways of 1,3,5-tri-*O*-acetyl-4,6-dideoxy-2-*O*-methyl-4-(*N*-methyl)acetamido)-mannitol-1-*d* (**a**) and 1,2,5-tri-*O*-acetyl-4,6-dideoxy-3-*O*-methyl-4-(*N*-methyl)acetamido)-mannitol-1-*d* (**b**) obtained from the O-PS of *A. veronii* bv. *sobria* strain K557. Diagnostic primary and secondary fragment ions are indicated. The mass difference 42, 60, or 32 indicates loss of chetene, acetic acid, or methanol, respectively.

The methylation analysis of the O-PS by GLC-MS of partially methylated alditol acetates resulted in the identification of 4,6-dideoxy-3-*O*-methyl-4-(*N*-methyl)acetamidohexose (derived from 2-substituted Rha4N), and 4,6-dideoxy-2-*O*-methyl-4-(*N*-methyl)acetamidohexose (derived from 3-substituted Rha4N). The *EI* (electron impact) mass spectrum (Figure 4) of 4,6-dideoxy-2-*O*-methyl-4-(*N*-methyl)acetamidomannose was characterized by the presence of intense ion peaks at *m/z* 118 (C-1 ÷ C2 fragment), 275 (C-1 ÷ C4 fragment), and 172 (C-4 ÷ C6 fragment), and allowed distinguishing this derivative from 4,6-dideoxy-3-*O*-methyl-4-(*N*-methyl)acetamidomannose, whose *EI* mass spectrum contained ions at *m/z* 190, 275, and 172 characteristic of the C-1 ÷ C3, C-1 ÷ C4, and C-4 ÷ C6 primary fragments, respectively.

**Figure 5:**
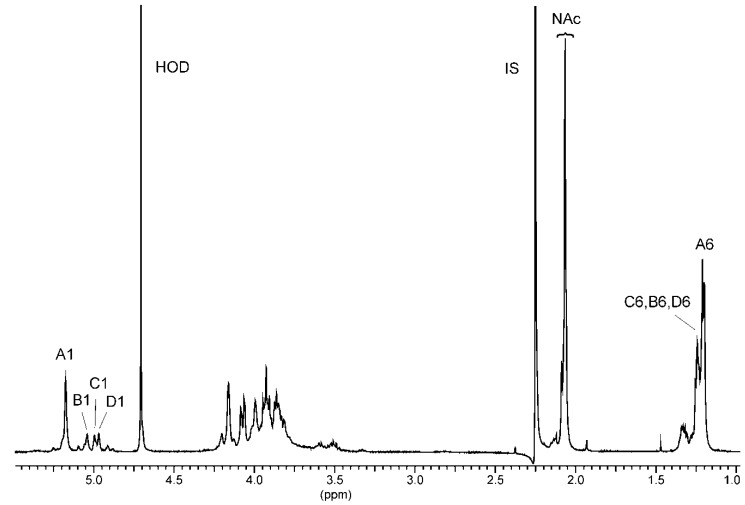
^1^H NMR spectrum of the O-PS of *A. veronii* bv. *sobria* strain K557. The spectrum was recorded in D_2_O at 32 °C at 500 MHz. Capital letters and Arabic numerals refer to atoms in the sugar residues denoted as shown in Table 3. NAc—*N*-acetyl groups, IS—acetone as an internal standard (δ_H_ 2.225), asterisk—free acetic acid.

The low-field region of the ^1^H NMR spectrum of the O-polysaccharide (Figure 5) contained one major and three minor signals for anomeric protons at δ 5.18, 5.04, 5.00, and 4.97. The high-field region of the spectrum included signals for *N*-acetyl groups at δ 2.07, CH_3_-C groups of 6-deoxy sugars in the range of δ 1.21–1.24. The ^1^H and ^13^C resonances of the O-PS of *A. veronii* bv. *sobria* K557 were assigned using 2D homonuclear ^1^H,^1^H DQF-COSY, TOCSY, NOESY, heteronuclear ^1^H,^13^C HSQC, and HMBC experiments. The ^1^H and ^13^C NMR data are collected in Table 3. 

**Figure 6:**
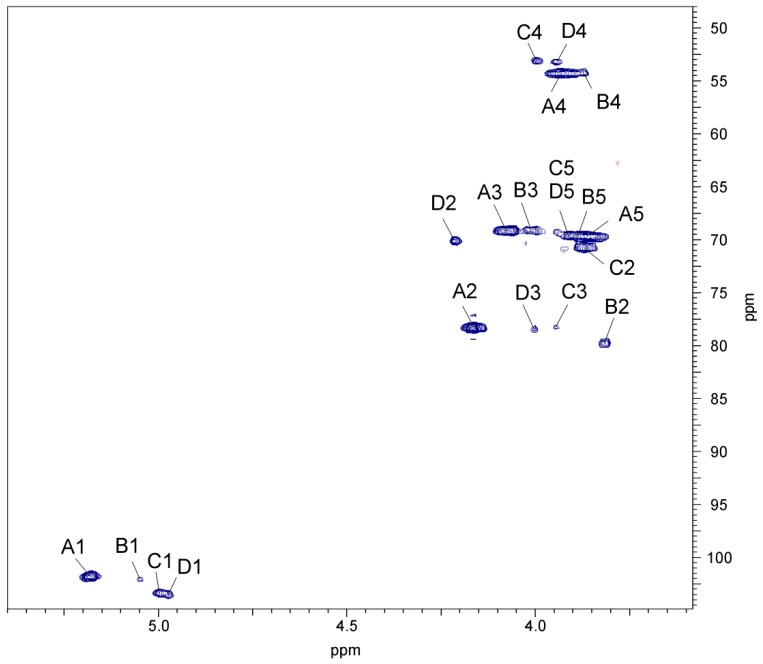
Part of a ^1^H,^13^C HSQC spectrum (500 x 125 MHz) of the O-PS of *A. veronii* bv. *sobria* strain K557. The spectrum was recorded at 32 °C in D_2_O as a solvent. Capital letters and Arabic numerals refer to atoms in sugar residues denoted as shown in Table 3.

The ^1^H,^1^H TOCSY, and DQF-COSY spectra revealed one major and three minor spin systems for monosaccharide residues, which were labelled **A**–**D** in the order of the decreasing chemical shifts of their anomeric protons. The high-field positions of H-6 (δ 1.21–1.24) and C-6 (δ 18.2) resonances and the values of vicinal coupling constants ^3^*J*_1,2_ (~2 Hz), ^3^*J*_3,4_ (8.5 Hz), ^3^*J*_4,5_ (9 Hz), and ^3^*J*_5,6_ (6 Hz) characteristic for *manno*-pyranose allowed assigning all the **A**, **B**, **C**, and **D** spin systems (H-1/C-1 cross-peaks at δ 5.18/101.7, 5.04/102.1, 5.0/103.3, and 4.97/103.5, respectively) to Rha4NAc residues. The latter conclusion was confirmed by the correlation signals at δ 3.93/54.3 (the major one), 3.87/54.3, 3.94/53.2, and 4.00/53.2 observed in the ^1^H,^13^C HSQC spectrum (Figure 6), which indicated that the O-PS contains *N*-acetamido sugar. 

In the TOCSY spectrum, at the H-1 coordinate, the cross-peaks with H-2-H-5 were visible for Rha4NAc **A**, but only one cross-peak with H-2 for Rha4NAc **B**, and two ones with H-2 and H-3 for Rha4NAc **C** and **D**. In turn, starting from the H-2 proton signal, cross-peaks with H-3-H-6 were visible for all spin systems; however, some signals overlapped. The ^1^H,^1^H COSY spectrum allowed unambiguous differentiation between protons within the spin system **A** and only partly resolved the cross-peaks for the Rha4NAc **B**, **C** and **D**. The difficulties in the assignment of the H-3, H-4, and H-5 of Rha4NAc **B**, **C** and **D** were overcome in the ^1^H,^13^C HMBC and ^1^H, ^13^C HSQC experiments. 

The ^13^C NMR resonances of e.g., Rha4NAc **C** were assigned by the long-range H-6/C-4 and H-6/C-5 correlations at δ 1.24/53.2 and 1.24/69.5, and then the C-4/H-3 and C-4/H-2 correlations at δ 53.2/3.93 and 53.2/3.87, respectively, in the ^1^H,^13^C HMBC spectrum. In the ^1^H,^13^C HSQC spectrum, the cross-peak of the proton at the nitrogen-bearing carbon to the corresponding carbon at δ 4.00/53.2 was assigned to the H-4/C-4 correlation of Rha4NAc **C**. Moreover, in the ^1^H,^13^C HMBC spectrum, correlations of the anomeric proton with carbons C-2 and C-5 were found, and then the proton resonances were assigned from the ^1^H,^13^C HSQC spectrum. Similar long-range correlations were searched during identification of H-3, H-4 and H-5 proton signals of Rha4NAc **B** and **D**. The chemical shifts of the C-2 signal of Rha4NAc **B** and the C-3 signals of Rha4NAc **C** and **D** were confirmed after consideration of the methylation analysis data. 

The α-configuration of all Rha4NAc residues was inferred from the relatively high-field position of the C-5 signals at δ 69.5–69.7 for the residues in the O-polysaccharides, compared with δ 68.6 and δ 72.4 for α-Rha4NAc and β-Rha4NAc, respectively [44,45]. 

The correlation signals between H-4 of all the Rha4NAc residues and carbonyl group signals at δ_C_ 176.0 and between the latter and methyl proton signals at δ_H_ 2.07, which were observed in the ^1^H,^13^C HMBC spectrum, confirmed that the residues building the O-PS were *N*-acetylated. 

The positions of glycosylation were determined by downfield shifted signals of carbon atoms C-2 for residues Rha4NAc **A** (δ 78.3) and **B** (δ 79.8), and C-3 for Rha4NAc **C** (δ 78.3) and **D** (δ 78.4), compared with their positions in the spectra of the corresponding non-substituted monosaccharides [44,45,46]. 

**Figure 7:**
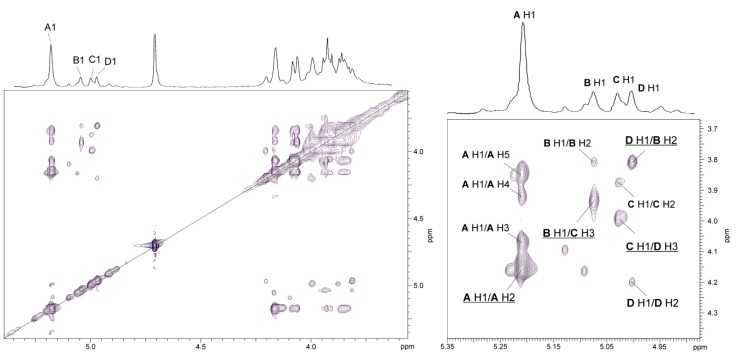
Parts of ^1^H,^1^H NOESY and ^1^H NMR (insert) spectra of the O-PS of *A. veronii* bv. *sobria* strain K557. The map shows NOE contacts between anomeric protons and protons at the glycosidic linkages (underlined). Some other H/H correlations are depicted as well. Capital letters and Arabic numerals refer to atoms in the sugars denoted as shown in Table 3.

In the NOESY spectrum (Figure 7), intraresidue H-1/H-2 and interresidue H-1/H-5 correlations at δ 5.18/4.16 and δ 5.18/3.85, respectively, typical of α-(1→2)-linked sugars with the *manno* configuration, indicated that the Rha4NAc **A** residues constitute the main O-polysaccharide (O-PS 1) being the homopolymer [47]. In turn, in the spectrum inter-residue NOE contacts were observed for protons of residues **B**→**C**, **C**→**D**, and **D**→**B**. The following correlations between the anomeric protons and protons at the linkage carbons: Rha*p*4NAc **B** H-1/Rha*p*4NAc **C** H-3 at δ 5.04/3.93; Rha*p*4NAc **C** H-1/Rha*p*4NAc **D** H-3 at δ 5.00/3.99; Rha*p*4NAc **D** H-1/Rha*p*4NAc **B** H-2 at δ 4.97/3.81 demonstrated that the other O-polysaccharide (O-PS 2) is a heteropolymer with a trisaccharide repeating unit (Figure 7, Table 3). 

**Figure 8:**
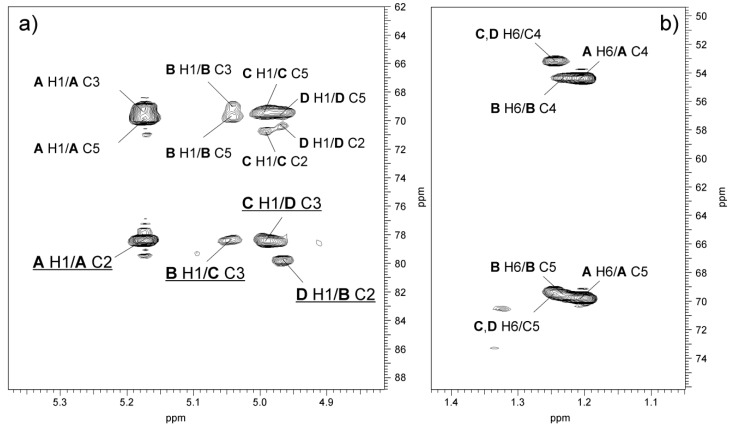
Regions of the ^1^H,^13^C HMBC spectrum of the O-PS of *A. veronii* bv. *sobria* strain K557. The maps show heteronuclear correlations for (**a**) anomeric protons, and (**b**) H-6 protons. Interresidue correlations between anomeric protons and carbons at the glycosidic linkages are underlined. Some other H/C correlations are depicted as well. Capital letters and Arabic numerals refer to protons or carbons in the sugar residues denoted as shown in Table 3.

The sequence of monosaccharides in the repeating unit of O-PS 2 was confirmed, in the ^1^H,^13^C HMBC spectrum (Figure 8), by the following inter-glycosidic cross-peaks: **B** H-1/**C** C-3 at δ 5.04/78.3; **C** H-1/**D** H-3 at δ 5.00/78.4; **D** H-1/**B** H-2 at δ 4.97/79.8. 

In conclusion, the O-antigen of *A. veronii* bv. *sobria* K557, O6 consists of two structurally different O-polysaccharides, both containing only l-Rha4NAc residues. One of them (O-PS 1) is an α-(1→2)-linked homopolymer of l-Rha4NAc. The other polysaccharide, O-PS 2, is built up of trisaccharide repeating units composed of one α-(1→2)-, and two α-(1→3)-linked l-Rha*p*4NAc residues. 

As judged by the comparison of the integral intensities of the H-1 proton signals, the content of O-PS 1 in the O-antigen of *A. veronii* bv. *sobria* K557 was predominant and was estimated at approximately 64%. 

Based on the composition and methylation analyses as well as the NMR data, the following structures of the O-polysaccharides have been established: 

O-PS 1    →2)-α-l-Rha*p*4NAc-(1→

          **A**

O-PS 2    →2)-α-l-Rha*p*4NAc-(1→3)-α-l-Rha*p*4NAc-(1→3)-α-l-Rha*p*4NAc-(1→

           **B**          **C**        **D**

**Figure 9:**
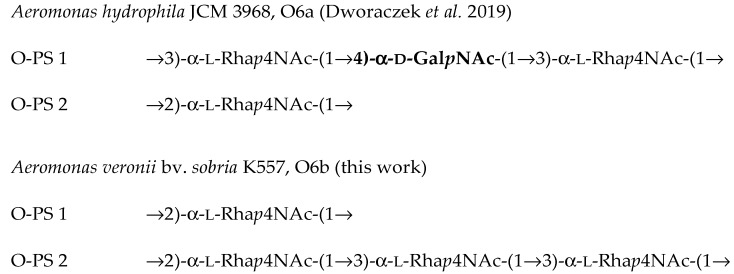
Structures of the O6a and O6b antigens repeating units. The additional sugar residue characteristic for serotype O6a and not defined in serotype O6b is shown in bold type.

The structure of the *A. veronii* bv. *sobria* K557 O-antigen is similar but not identical to the recently published O-PS of *Aeromonas hydrophila* JCM 3968, O6, which consisted of two structurally different O-polysaccharides as well (Figure 9). One of them (O-PS 1) was a heteropolymer built up of trisaccharide repeating units composed of one 4-substituted α-d-Gal*p*NAc and two α-(1→3)-linked l-Rha*p*4NAc residues. The other polysaccharide, O-PS 2, was an α-(1→2)-linked homopolymer of l-Rha4NAc [37]. 

The serological results (Western blotting and ELISA) allowed recognition of structural determinants that are most probably responsible for antibody binding (putative epitopes). The occurrence of common α-l-Rha*p*4NAc-(1→2)-α-l-Rha*p*4NAc and α-l-Rha*p*4NAc-(1→3)-α-l-Rha*p*4NAc disaccharides is sufficient for providing cross-reactivity between the *A. hydrophila* JCM 3968 O6 antiserum and the LPS of *A. veronii* bv. *sobria* K557 and a similar reaction in an opposite system. 

On the other hand, an additional epitope (epitopes) most probably related to a 4-substituted α-d-Gal*p*NAc residue or a →4)-α-d-Gal*p*NAc-(1→3)-α-l-Rha*p*4NAc disaccharide fragment, which has not been found in the O-antigen studied here, determines the specificity of the *A. hydrophila* JCM 3968 O6 serotype. This putative epitope (epitopes) seems to play an important role in the immunospecificity of the reference O6 antiserum (Figure 9). 

Based on the data obtained, we suggest the division of the O6 serogroup into two subgroups: serogroup O6a for strains that share α-l-Rha*p*4NAc-(1→2)-α-l-Rha*p*4NAc and α-l-Rha*p*4NAc-(1→3)-α-l-Rha*p*4NAc disaccharides and an additional →4)-α-d-Gal*p*NAc-associated epitope, and the other serogroup O6b deprived of the latter structural fragment in the O-antigen, with strain *A. veronii* bv. *sobria* K557 as a representative of the new subgroup. 

## 3. Discussion

Almost every year, health disorders in freshwater fish are recorded on many farms in Poland. Although the development of a particular fish disease depends largely on climate conditions prevailing in a given zone and region, infections caused by *Aeromonas* spp. are the most common among bacterial fish diseases. As demonstrated recently by data collected during the last several years in Poland by Pękala-Safińska, health disorders caused by *Aeromonas* species were mostly observed in carp (*Cyprinus carpio* L.) and were usually manifested by skin lesions (MAI) in the form of ulceration as well as fish mortalities [48]. 

Previous studies performed on 558 isolates of mesophilic *Aeromonas* collected during 5 years in Polish culture facilities, among which 427 isolates were obtained from common carp and 121 from rainbow trout, revealed predominant occurrence of *A. veronii* bv. *sobria*, *A. sobria* and *A. salmonicida* species. In turn, *A. veroni* bv. *sobria A. bestiarum* and *A. salmonicida* were most frequently identified in both carp and trout samples; *A. veroni* bv. *sobria* was one of the dominant species only among carp isolates, and which is worth emphasizing, almost 80% of these isolates were classified as virulent [28,36]. Serological typing of all collected isolates using 44 antisera of the NIH scheme extended by 20 provisional serogroups for selected Polish isolates showed that O6 was one of the five most commonly identified serogroups, especially among carp isolates, and the other ones were O11, PGO1, O16, and O18. The report mentioned above also showed that, with the use of the antisera for serogroups from O45 to O96, there was little possibility for *Aeromonas* typing, since these groups occur rather rarely among fish isolates, especially in Polish aquacultures. In turn, when the 44 antisera of the NIH scheme were used, about 53% isolates were positively classified to appropriate somatic serotypes and this increased to about 88% when the NIH collection was extended by the sera for provisional serogroups of Polish origin [28,36]. Therefore, to obtain the most positive serotyping results, the postulate to include new provisional antisera against strains occurring in a given area and thus to extend the collection of antisera seems reasonable. 

The inland aquaculture sector in Poland is mainly based on the culture of two species of freshwater fish - carp (49% of total production in 2015) and rainbow trout (38% of total production in 2015) [49]; thus, the lack of a commercially available vaccine dedicated to carp seems to be alarming. The most effective prevention of fish disease should involve immunoprophylaxis based on auto-vaccines chosen according to the needs of culture facilities and prepared from bacterial strains isolated from the fish or even the entire region. Auto-vaccines are confirmed to be highly effective against conditionally pathogenic microorganisms like *Aeromonas* sp*., Yersinia ruckeri*, and *Pseudomonas* sp. [50,51,52,53]. According to recently published studies, a vaccine containing whole cells and LPS of *Aeromonas* sp. seems to protect fish against MAS disease [54,55,56]. However, to avoid a failure of implemented prophylactic programs based on auto-vaccination, the serological and structural similarities and differences in O-chain polysaccharides in various serogroups and strains, which contribute to the immunospecificity of *Aeromonas*, should be carried out [57]. 

Here, the immunochemical investigation of LPS, especially the O-specific polysaccharide of *A. veronii* bv. *sobria* strain K557 were performed. SDS-PAGE and Western blotting revealed that the LPS-glycoforms of *A. veronii* bv*. sobria* K557 contained shorter O-chains than those of *A. hydrophila* JCM 3968; however, these molecules represented S-LPS species rather with the prevalence of the intermediate length O-antigen chains. In a recent study, Osawa et al. reported that the lengths of the *E. coli* O157 antigen could be modulated by *wzz* gene mutations and it has been shown that strains with long, intermediate, and short O-antigens vary in sensitivity to serum complement. The greater resistance of strains with intermediate and/or long length O-antigen chains to serum complement lysis than those with short O-chains is likely to have been optimized for pathogenesis during evolution [58]. 

Moreover, we established the structure of the O-antigen of *A. veronii* bv*. sobria* strain K557, and found that it consisted of two different O-polysaccharides. The serological studies indicated that the O-PS of *A. veronii* bv. *sobria* strain K557 is closely related but not identical to the O-antigen of *A. hydrophila* strain JCM 3968, which is a reference strain to *Aeromonas* serogroup O6. Another peculiar characteristic of both O-PSs is the presence of 4-amino-4,6-dideoxy-l-mannose (l-Rha4N, l-perosamine), quite an unusual amino sugar, for the first time identified as a compound building the O-antigen of *A. hydrophila* strain JCM 3968, O6 [37]. This is the second work that shows the occurrence of l-perosamine as a component of bacterial O-polysaccharides. 

The chemical and mass spectrometry analyses of the phenol-soluble LPS of *A. veronii* bv. *sobria* strain K557 demonstrated the LPS glycoforms had hexaacylated lipid A species with a conserved architecture and a backbone composed of 1,4′-bisphosphorylated-β-(1→6)-linked-d-GlcN disaccharide. The residues of 3-hydroxytetradecanoate were predominant among fatty acids, similarly as previously reported for *A. hydrophila* [37]. However, some differences were found in the acylation profile of lipid A species, in comparison to those of *A. bestiarum* [59]. Amongst the ester-linked saturated fatty acids, not only dodecanoic (12:0) but also tetradecanoic (14:0) residues were found. The latter fatty acids were also detected in the lipid A of *A. veronii* strain Bs19, O16 [60].

The compositional analysis of the core oligosaccharide revealed two isomers of heptose (d,d-Hep and l,d-Hep), and MALDI-TOF MS confirmed that the core decasaccharide, with the following composition: Hep_6_Hex_2_HexN_1_Kdo_1_P_1_, has a structure shared by the LPS core regions of the *A. hydrophila* and *A. bestiarum* species [37,59]. Interestingly, the mass spectrum of the core OS of *A. hydrophila* JCM 3968, which was isolated from R-LPS molecules after mild acid hydrolysis and separation by gel-permeation-chromatography, showed an ion at *m*/*z* 1856.59, which corresponded to the dephosphorylated core variant with the composition Hep_6_Hex_2_HexN_1_Kdo*_anh_*. On the other hand, the structure of the core OS in the SR-LPS molecules of *A. hydrophila* JCM 3968 was slightly different: Hep_6_Hex_1_HexN_1_HexNAc_1_Kdo_1_P_1_ [37]. In these species, the O-antigen was linked to the GalNAc residue, whereas in the rough R-LPS glycoforms, the galactose appeared to be a terminal outer core sugar, similarly as has been established for the core OS of the rough mutant strain of *A. hydrophila* AH-3, O34 [39]. 

In conclusion, the immunochemical studies of LPS, which is a glycolipid characterized by high heterogeneity amongst *Aeromonas* sp. bacteria, may facilitate selection of vaccine strains suitable for immunoprophylaxis of MAI/MAS diseases. The O-antigen of *A. veronii* bv*. sobria* strain K557, serotype O6, studied here is composed of two O-polysaccharides, both containing a unique sugar 4-amino-4,6-dideoxy-l-mannose (*N*-acetyl-l-perosamine, l-Rha*p*4NAc). The major O-polysaccharide (O-PS 1) is built up of an α1→2 linked of l-Rha*p*4NAc, whereas the other one, O-PS 2, has trisaccharide repeating units composed of α1→2 and α1→3 linked l-Rha*p*4NAc residues. The serological studies confirmed the structural analyses and showed that the O-antigens of *A. veronii* bv*. sobria* K557, i.e., the strain isolated from the common carp during an outbreak of MAI/MAS on a Polish fish farm, and *A. hydrophila* JCM 3968, represent the same *Aeromonas* serogroup O6 and are closely related but not identical. The recently studied O-antigen of *A. hydrophila* JCM 3968, O6 consisted of two structurally different O-polysaccharides. One of them was a heteropolymer built up of trisaccharide repeating units composed of one α-d-Gal*p*NAc and two α-(1→3)-linked l-Rha*p*4NAc residues. The other O-polysaccharide was an α-(1→2)-linked homopolymer of l-Rha4NAc. The consideration of the serological results in view of the known O-antigen structures enabled recognition of domains that could be responsible for antibody binding (putative epitopes). The occurrence of common α-l-Rha*p*4NAc-(1→2)-α-l-Rha*p*4NAc and α-l-Rha*p*4NAc-(1→3)-α-l-Rha*p*4NAc disaccharides is sufficient for providing cross-reactivity between *A. hydrophila* JCM 3968 O6 antiserum and the LPS of *A. veronii* bv. *sobria* 557, and *vice versa*. On the other hand, the additional epitope related to a 4-substituted α-d-Gal*p*NAc residue, which has not been found in the O-antigen studied here, determines the specificity of the *A. hydrophila* JCM 3968 O6-serotype. This putative epitope seems to play an important role in the immunospecificity of the reference O6 antiserum. 

Therefore, based on the data obtained, we suggest division of the O6 serogroup into two subgroups. The serogroup O6a includes strains that share α-l-Rha*p*4NAc-(1→2)-α-l-Rha*p*4NAc and α-l-Rha*p*4NAc-(1→3)-α-l-Rha*p*4NAc disaccharides and an additional →4)-α-d-Gal*p*NAc-associated epitope. *A. veronii* bv. *sobria* strain K557 is a representative of the other subgroup O6b and its O-antigen is deprived of the latter structural fragment. 

## 4. Materials and Methods 

### 4.1. Bacterial Strain, Cultivation Conditions, and Isolation of LPS

*Aeromonas veronii* strain K557 was isolated from a common carp during an outbreak of motile aeromonad infection/motile aeromonad septicemia (MAI/MAS) on a Polish fish farm. The isolate was identified to the species level by restriction analysis of 16S rRNA gene amplified by polymerase chain reaction [28] and classified as *Aeromonas veronii* bv. *sobria* because of the positive reaction for arginine dihydrolase, and negative reactions for ornithine decarboxylase and aesculin hydrolysis [30,31]. Based on virulence-associated markers (hemolytic, gelatinolytic, and caseinolytic activities), strain K557 was classified as virulent for fish. For the LPS analysis, *A. veronii* bv. *sobria* K557 bacterium was cultivated with shaking (120 rpm) on tryptic soy broth (TSB) for 72 h at 28 °C. The cells were harvested by low-speed centrifugation (8000× *g*, 20 min). The recovered bacterial cell pellet was washed twice with 0.85% saline and once more with distilled water. 

The bacterial cells (5 g dry mass) were digested with lysozyme, RNAse, and DNAse (24 h, 1 mg/g) and then with Proteinase K (36 h, 1 mg/g) in 50 mM phosphate buffer (pH 7.0) containing 5 mM MgCl_2_. The suspension was dialyzed against distilled water and freeze-dried. The digested cells were extracted three times with aqueous 45% phenol at 68 °C, [38] and the separated layers were dialyzed against tap and distilled water. LPS species recovered from the phenol phase were purified by ultracentrifugation at 105,000× *g* and freeze-dried to give a yield of 3.9% of dry bacterial cell mass. 

### 4.2. Degradation of LPS and Isolation of O-polysaccharide

The phenol-soluble S-LPS (110 mg) was hydrolyzed with aqueous 2.5% acetic acid at 100 °C for 3 h, and lipid A precipitate was removed by centrifugation. The supernatant was concentrated and then fractionated by GPC on a column (1.8 × 80 cm) of Sephadex G-50 fine (Pharmacia, Sweden) using 1% acetic acid as the eluent and monitoring with a differential refractometer (Knauer, Berlin, Germany). The yield of the O-PS fraction was 33% of the LPS mass subjected to hydrolysis. 

### 4.3. Chemical Analyses

For neutral and amino sugar analysis, the LPS and O-PS samples were hydrolyzed with 2 M CF_3_CO_2_H (100 °C, 4 h) or 10 M HCl for 30 min at 80 °C, respectively, and reduced with NaBD_4_; this was followed by acetylation with a 1:1 (*v*/*v*) mixture of acetic anhydride and pyridine (85 °C, 0.5 h). 

To release acidic sugar, LPS was dephosphorylated with 48% aqueous hydrofluoric acid, HF (4 °C, 18 h) and dried under vacuum over sodium hydroxide [40]. Methanolysis was performed with 1 M MeOH/HCl (85 °C, 1 h), and the sample was extracted twice with hexane. The methanol layer was concentrated and the residue was dried and acetylated. The monosaccharides were identified as alditol and aminoalditol acetates [61] as well as acetylated methyl glycosides by GLC-MS. 

For determination of the absolute configuration [42], the O-PS was subjected to 2-octanolysis (300 μL (*S*)-(+)-2-octanol or (*SR*)-(±)-2-octanol and 20 μL acetyl chloride, 100 °C, 3 h); the products were acetylated and analyzed by GLC-MS as above. A sample from the polysaccharide of *Citrobacter gillenii* O9a,9b [43] was used as the reference standard of d-Rha4N (D-perosamine). 

Methylation of the O-PS (1.0 mg) was carried out with methyl iodide in dimethyl sulfoxide in the presence of powdered sodium hydroxide [62]. The products were recovered by extraction with chloroform/water (1:1, *v*/*v*), hydrolyzed with 10 M HCl for 30 min at 80 °C, *N*-acetylated, and reduced with NaBD_4_. The partially methylated alditol acetates derivatives were analyzed by GLC-MS. 

For fatty acid analysis, a sample of the lipid A (1 mg) was subjected to methanolysis in 2 M methanolic HCl (85 °C, 12 h). The resulting fatty acid methyl esters were extracted with hexane and converted to their *O*-trimethylsilyl (*O*-TMS) derivatives, as described [63,64]. The methanol layer containing methyl glycosides was dried and acetylated with a pyridine-acetic anhydride mixture. The fatty acid derivatives and acetylated methyl glycosides were analyzed by GLC-MS as above. 

All the sample derivatives were analyzed on an Agilent Technologies 7890A gas chromatograph (Agilent Technologies, Wilmington, DE, USA) connected to a 5975C MSD detector (inert XL EI/CI, Agilent Technologies, Wilmington, DE, USA). The chromatograph was equipped with an HP-5MS capillary column (Agilent Technologies, 30 m × 0.25 mm, flow rate of 1 mL/min, He as carrier gas). The temperature program for all the derivatives was as follows: 150 °C for 5 min, then 150 to 310 °C at 5 °C/min, and the final temperature was maintained for 10 min. 

### 4.4. NMR Spectroscopy

An O-PS sample was deuterium-exchanged by freeze-drying with D_2_O and then examined in 99.98% D_2_O using acetone as an internal standard (δ_H_ 2.225, δ_C_ 31.45). 1D and 2D NMR spectra were recorded at 32 °C on a 500 MHz NMR Varian Unity Inova instrument (Varian Associates, Palo Alto, CA, USA) using Varian software Vnmrj V. 4.2 rev. (Agilent Technologies, Santa Clara, CA, USA). The following homonuclear and heteronuclear shift-correlated two-dimensional experiments were conducted for signal assignments and determination of the sugar sequence: ^1^H,^1^H DQF-COSY, ^1^H,^1^H TOCSY,^1^H,^1^H NOESY, ^1^H,^13^C HSQC, and ^1^H,^13^C HMBC. The mixing time was set to 100 and 200 ms in the TOCSY and NOESY experiments, respectively. The ^1^H,^13^C HSQC experiment with CRISIS based multiplicity editing was optimized for a coupling constant of 146 Hz. The heteronuclear multiple-bond correlation (HMBC) experiment was optimized for *J*_H,C_ = 7 and 5 Hz, with 2-step low-pass filter 130 and 165 Hz to suppress one-bond correlations. 

### 4.5. MALDI-TOF Mass Spectrometry (MS)

LPS was analyzed with matrix-assisted laser desorption/ionization time-of flight (MALDI-TOF) mass spectrometry (MS) using a Waters SYNAPT G2-*Si* HDMS instrument (Waters Corporation, Milford, MA, USA) equipped with a 1 kHz Nd:YAG laser system. Acquisition of the data was performed using MassLynx software version 4.1 SCN916 (Waters Corporation, Wilmslow, UK). Mass spectra were assigned with a multi-point external calibration using red phosphorous (Sigma-Aldrich, St. Louis, MO, USA) and recorded in the negative ion mode. An LPS sample (at a concentration of 10 µg/µL) was suspended in a water/methanol (1:1, *v*/*v*) solution containing 5 mM EDTA and then dissolved by ultrasonication. After desalting with some grains of cation exchange beads (Dowex 50WX8-200; Sigma-Aldrich, St. Louis, MO, USA), one microliter of the sample was transferred onto a well plate covered with a thin matrix film and allowed to dry at room temperature. The matrix solution was prepared from 2’,4’,6’-trihydroxyacetophenone (THAP) (200 mg/mL in methanol) mixed with nitrocellulose (15 mg/mL) suspended in 2-propanol/acetone (1:1, *v*/*v*) in proportion of 4:1 (*v*/*v*), according to the published method [65]. 

### 4.6. SDS-PAGE

LPS preparations were separated in 12.5% SDS-Tricine polyacrylamide electrophoresis gel and bands were visualized by silver staining after oxidation with periodate according to the published method [66]. 

### 4.7. Serological Studies

Polyclonal O-antisera against *A. hydrophila* JCM 3968, serogroup O6 and *A. veronii* bv. *sobria* strain K557 were obtained by immunization of New Zealand white rabbits with heat-inactivated bacteria according to the published procedure [28]. Rabbits were acclimatized in the animal facility of the National Veterinary Research Institute (Puławy, Poland), and all the experiments were performed according to the procedures approved by the local ethical committee (The Second Local Ethical Committee on Animal Testing in Lublin, the permission number 48/2012). 

Western blots with rabbit antisera were performed after transferring SDS-PAGE-separated LPS profiles to Immobilon P (Millipore, St. Louis, MO, USA). The primary antibodies were detected using alkaline phosphatase-conjugated goat anti-rabbit antibodies (Sigma, St. Louis, MO, USA). Blots were developed with nitroblue tetrazolium and 5-bromo-4-chloro-3-indolylphosphate toluidine (Sigma) for 5 min, as described elsewhere [67].

The ELISA was performed as described previously [68] with some modifications, namely: 1–2 μg of the studied LPS per well were coated on flat-bottom Nunc-Immuno plates; the reaction was developed using rabbit-IgG specific peroxidase-conjugated goat antibodies (Jackson ImmunoResearch, West Grove, PA, USA); the final absorbance (A_405_) was read with the help of a Multiskan Go microplate reader (Thermo Fisher Scientific USA, Vantaa, Finland). 

Adsorption of antisera was carried out using wet masses of bacterial cells washed in PBS (phosphate-buffered saline). Bacterial mass (100 μL) was suspended in 1 mL of serum diluted 1:50 in PBS. After 0.5 h incubation on ice, the cells were removed by centrifugation and the process was repeated two or three more times [69]. 

## Figures and Tables

**Table 1:** Reactivity (reciprocal titers) of the studied antisera (intact or adsorbed) with the LPS preparations from the *A. hydrophila* JCM 3968 and *A. veronii* bv*. sobria* K557 strains.

Serum Specific to Strain		JCM 3968 LPS	K557 LPS
JCM 3968	intact	1,024,000	64,000
	K557 adsorbed	256,000	<1000
K557	intact	64,000	64,000
	JCM 3968 adsorbed	<1000	<1000

**Table 2:** Composition of the main species present in the negative ion MALDI-TOF mass spectrum of the LPS of *A. veronii* bv*. sobria* strain K557.

[M − H]^−^ Observed	[M − H]^−^ Calculated	M Monoisotopic	Assigned Composition
1768.178	1768.181	1769.188	HexN_2_P_2_[14:0(3-OH)]_4_(12:0)_2_
1796.194	1796.139	1797.146	HexN_2_P_2_[14:0(3-OH)]_3_[*i*15:0(3-OH)](12:0)_2_
1824.224	1824.243	1825.250	HexN_2_P_2_[14:0(3-OH)]_4_(14:0)_2_
1830.535	1830.626	1831.633	Hep_6_Hex_2_HexN_1_Kdo-COO
1874.542	1874.616	1875.624	Hep_6_Hex_2_HexN_1_Kdo
1910.519	1910.593	1911.600	[Hep_6_Hex_2_HexN_1_KdoP]-COO
1954.556	1954.583	1955.599	Hep_6_Hex_2_HexN_1_KdoP

**Table 3:** ^1^H (500 MHz) and ^13^C NMR (125 MHz) data (δ, ppm) for the O-PS of *A. veronii* bv. *sobria* strain K557.

Sugar Residue		Chemical Shifts (δ, ppm)					
		H-1 C-1	H-2 C-2	H-3 C-3	H-4 C-4	H-5 C-5	H-6 C-6
→2)-α-l-Rha*p*4NAc-(1→	A	5.18	4.16	4.07	3.93	3.85	1.21
		101.7	78.3	69.2	54.3	69.7	18.2
→2)-α-l-Rha*p*4NAc-(1→	B	5.04	3.81	4.01	3.87	3.91	1.22
		102.1	79.8	69.2	54.3	69.6	18.2
→3)-α-l-Rha*p*NAc-(1→	C	5.00	3.87	3.93	4.00	3.92	1.24
		103.3	70.7	78.3	53.2	69.5	18.2
→3)-α-l-Rha*p*4NAc-(1→	D	4.97	4.20	3.99	3.94	3.92	1.24
		103.5	70.2	78.4	53.2	69.5	18.2

Chemical shifts for NAc are δ_H_ 2.07 and δ_C_ 23.4 (CH_3_) and 176.0 (CO).
